# A New Algebraic Solution for Acoustic Emission Source Localization without Premeasuring Wave Velocity

**DOI:** 10.3390/s21020459

**Published:** 2021-01-11

**Authors:** Zilong Zhou, Riyan Lan, Yichao Rui, Longjun Dong, Xin Cai

**Affiliations:** School of Resources and Safety Engineering, Central South University, Changsha 410083, China; zlzhou@csu.edu.cn (Z.Z.); lanriyancsu@csu.edu.cn (R.L.); lj.dong@csu.edu.cn (L.D.); xincai@csu.edu.cn (X.C.)

**Keywords:** acoustic emission (AE), source localization, wave velocity, sum of squared residuals, algebraic solution, time-difference-of-arrival (TDOA)

## Abstract

The technique of acoustic emission (AE) source localization is critical for studying material failure mechanism and predicting the position of potential hazards. Most existing positioning methods heavily depend on the premeasured wave velocity and are not suitable for complex engineering practices where the wave velocity changes dynamically. To reduce the influence of measurement error of wave velocity on location accuracy, this paper proposes a new algebraic solution for AE source localization without premeasuring wave velocity. In this method, the nonlinear TDOA equations are established and linearized by introducing two intermediate variables. Then, by minimizing the sum of squared residuals of the linear TDOA equations with respect to the AE source coordinate and two intermediate variables separately, the optimal algebraic solution of the AE source coordinate in the least squares sense is obtained. A pencil-lead breaks experiment is performed to validate the positioning effectiveness of the proposed method. The results show that the new method improves the positioning accuracy by more than 40% compared with two pre-existing methods, and the minimum positioning accuracy of the proposed method can reach 1.12 mm. Moreover, simulation tests are conducted to further verify the location performance of the proposed method under different TDOA errors and the number of sensors.

## 1. Introduction

Acoustic emission (AE) source localization, as an important non-destructive testing technology, is widely used to study material failure mechanism and predict the position of potential hazards [1,2,3,4,5,6,7,8,9,10]. There are many traditional positioning methods, including Taylor-based methods [11], Inglada method [12], spherical-interpolation method [13], spherical-intersection method [14], and plane-intersection method [15]. The localization accuracy of these methods strongly depends upon the measurement precision of wave velocity [16]. However, it is very hard or impossible to determine the wave velocity exactly in real engineering practices due to the complex geological structures and dynamic variations in engineering environments. Specifically, due to the complex geological structures (e.g., fault, cavity, joint, fracture), the average wave velocities of each region are different, and the location of the real AE source is not necessarily within the range of the pre-determined wave velocity [17,18,19]. Moreover, as the construction operations progress, the engineering environment will change dynamically, causing stress adjustments and rock mass structure changes, in turn leading to changes in the regional average velocity. It was reported that the positioning error might be over 100 m when the existing positioning methods with premeasured wave velocity were applied to an underground mine with complex geological structures [20,21]. Furthermore, Dong et al. [22] compared the accuracy of the localization methods with and without the premeasured wave velocity by performing the virtual positioning test. Their results showed that the 1–5% floating of the wave velocity could result in large location errors. Therefore, there is an urgent need to eliminate the dependence on the wave velocity measurement to improve the accuracy of AE source localization.

In recent years, several localization methods that are free of premeasuring wave velocity have been developed [23]. For instance, Dong et al. [22] first proposed three non-linear mathematic functions for AE source localization without premeasuring wave velocity, in which the average wave velocity could be jointly inversed with the source coordinate. Ciampa and Meo [24] exploited Newton’s iterative method to calculate the coordinate of the AE source location and the average wave velocity. Dehghan Niri et al. [25] considered the uncertainties in arrival measurements and average wave velocity using the extended Kalman filter algorithm, which further improved the location accuracy. All these methods can avoid the influence of the measurement deviation of the average wave velocity and are more suitable for practical applications. However, the requirements of a proper initial location and velocity estimate that is close to the true solution are rarely met in situ practice, leading to the problem of no convergence or slow convergence [26]. This problem leads to restraint in the extensive application of the iterative methods. In comparison, the algebraic methods are more computationally attractive [27,28]. Kundu et al. [29] gave the algebraic solution of the AE source coordinate with unknown wave velocity based on specially arranged sensor clusters. This localization method is efficient, since the solving process can avoid the above-mentioned drawbacks caused by iterative algorithms. However, placing sensors in such a special array is difficult and time-consuming in most scenarios. To avoid the use of such sensor clusters, Li et al. [30] derived the algebraic solution of spatial source by using six randomly arranged sensors. Nevertheless, the use of only six sensors makes this method unsuitable for positioning problems with more sensors. To comprehensively utilize more sensor data, the localization method using the extended principle of spherical intersection (ESX) was proposed to locate the AE sources [31,32]. However, the cost function constructed in this method is not optimal, leading to low location accuracy, especially when the AE source approaches the sensor array [33,34]. Moreover, the priori solution of the wave velocity solved by a quadratic equation might cause the phenomena of no-solution and multi-solution. To address the above problems, Dong et al. [20,35] proposed the new method requiring initial positioning. In this method, the initial positioning was conducted first to obtain the initial AE source coordinates, and then they were fitted by the logistical distribution function. Zhou et al. [36] further improved this by using a more advanced kernel density estimator (KDE). However, the positioning results of this method are still not optimal, because there is no optimization ability in the initial positioning [37]. Moreover, the real-time performance of this method tends to be poor, because of the requirement of intensive initial positioning.

To further improve the positioning accuracy and efficiency, a new algebraic solution for AE source localization without premeasuring wave velocity is proposed in this study. First, the linear TDOA equations of unknown wave velocity are established, and the residuals of the linear equations are calculated. Second, the sum of squared equation residuals is minimized with respect to the source coordinate and two intermediate variables separately, and the least squares algebraic solution can be obtained efficiently. The pencil-lead breaks experiment and simulation analysis are performed to verify the improvement in the location performance of the proposed method.

## 2. Related Works

To overcome the disadvantage of premeasuring the wave velocity, as mentioned in Section 1, scholars have given a series of algebraic solutions of AE sources. Among them, the AE source localization method for unknown wave velocity system proposed by Li et al. [30] is one of the most popular localization methods. Most of the existing velocity-free methods are developed based on this method. The basic principle of the existing method is derived as follows.

First, assume that the AE source and six sensors are located at $o\left(x,\;y,\;z\right)$ and $s_{i}\left(x_{i},\;y_{i},\;z_{i}\right)$ (i=1, 2, 3, 4, 5, 6), respectively. According to the proportional relationship between distance and time traveled from source to sensors, the nonlinear governing equations of AE source coordinate can be constructed as

(1) $$\sqrt{{\left(x_{i}-x\right)}^{2}+{\left(y_{i}-y\right)}^{2}+{\left(z_{i}-z\right)}^{2}}\;=v\left(t_{i}-\tau \right),\;\;i=1,\;2,\;3,\;4,\;5,\;6$$

where $t_{i}$ is the arrival time detected by sensor $s_{i}$. The symbol τ represents the trigger time of the AE source, and v denotes the average wave velocity in the propagation path.

Square (1) to give a new expression

(2) $${\left(x_{i}-x\right)}^{2}+{\left(y_{i}-y\right)}^{2}+{\left(z_{i}-z\right)}^{2}\;=v^{2}{\left(t_{i}-\tau \right)}^{2}$$

Subtract the equation of i=1 from the others (i=2, 3, 4, 5, 6), and the following equations can be given as

(3) $$2x\left(x_{i}-x_{1}\right)+2y\left(y_{i}-y_{1}\right)+2z\left(z_{i}-z_{1}\right)+2v^{2}\tau \left(t_{i}-t_{1}\right)+v^{2}\left(t_{1}^{2}-t_{i}^{2}\right)=l_{i,1}$$

where $l_{i,1}=\left(x_{i}^{2}-x_{1}^{2}\right)+\left(y_{i}^{2}-y_{1}^{2}\right)+\left(z_{i}^{2}-z_{1}^{2}\right)$ and i=2, 3, 4, 5, 6. 

Replacing $v^{2}\tau$ and $v^{2}$ with *S* and *V,* respectively, (3) becomes the following linear equations

(4) $$2x\left(x_{i}-x_{1}\right)+2y\left(y_{i}-y_{1}\right)+2z\left(z_{i}-z_{1}\right)+2S\left(t_{i}-t_{1}\right)+V\left(t_{1}^{2}-t_{i}^{2}\right)=l_{i,1},\;\;i=2,\;3,\;4,\;5,\;6$$

Equation (4) can also be reconstructed as

(5) AS=B

where $\begin{array}{l} A=\left[\begin{array}{l} 2(x_{2}-x_{1})\;\;\;2(y_{2}-y_{1})\;\;\;2(z_{2}-z_{1})\;\;\;2(t_{2}-t_{1})\;\;\;t_{1}^{2}-t_{2}^{2}\\ 2(x_{3}-x_{1})\;\;\;2(y_{3}-y_{1})\;\;\;2(z_{3}-z_{1})\;\;\;2(t_{3}-t_{1})\;\;\;t_{1}^{2}-t_{3}^{2}\\ 2(x_{4}-x_{1})\;\;\;2(y_{4}-y_{1})\;\;\;2(z_{4}-z_{1})\;\;\;2(t_{4}-t_{1})\;\;\;t_{1}^{2}-t_{4}^{2}\\ 2(x_{5}-x_{1})\;\;\;2(y_{5}-y_{1})\;\;\;2(z_{5}-z_{1})\;\;\;2(t_{5}-t_{1})\;\;\;t_{1}^{2}-t_{5}^{2}\\ 2(x_{6}-x_{1})\;\;\;2(y_{6}-y_{1})\;\;\;2(z_{6}-z_{1})\;\;\;2(t_{6}-t_{1})\;\;\;t_{1}^{2}-t_{6}^{2} \end{array}\right] \end{array}$, $S=\left[\begin{array}{c} x\\ y\\ z\\ S\\ V \end{array}\right],$ and $B=\left[\begin{array}{c} l_{2,1}\\ l_{3,1}\\ l_{4,1}\\ l_{5,1}\\ l_{6,1} \end{array}\right].$

By solving the above-determined linear equations (i.e., the number of equations is equal to the number of unknowns), the AE source coordinate (x, y,z), wave velocity v, and trigger time τ can be determined.

The above method is used under the condition that the number of sensors is six. However, there always are more sensors available in the engineering practice. From the viewpoint of error control, one method should use as many sensors as possible when performing source localization. There are two main reasons for this. First, the positioning accuracy is highly dependent on the geometry array of the AE sensors. Generally speaking, the more sensors are used, the better the sensor array is arranged [37]. Second, when more sensors are used, datasets will be statistically more reliable. Therefore, to obtain more accurate location results, it is essential to make full use of all the available sensors. Dong et al. [20,35] proposed the multi-sensor localization methods by using the prior logistic distribution function to fit the initial AE source coordinates, which further improved the location accuracy. The results showed that the absolute distance errors of three events in Dongguashan Mine were 6.85, 16.09, and 23.01 m, respectively, which are lower than the errors of the STT method (12.90, 16.17, 24.90 m) and TT method (10.99, 20.32, 32.43 m). However, the actual engineering conditions are complex, and a precise prior distribution of AE source coordinates is difficult to obtain. Moreover, the AE source coordinates *x*, *y*, and *z* are fitted separately, disregarding the correlation among them. Zhou et al. [36] further improved this by utilizing the principle of the tri-variate kernel density estimator, which is referred to as the KDE method in this paper. The KDE method considered the correlation among the AE source coordinates *x*, *y*, and *z*, and a more reasonable density function was obtained by extracting the information from the data themselves rather than assuming a classic distribution in advance. The KDE method was verified to give a better estimate of the probability density of the AE source coordinates, and the location accuracy was further improved, especially when outliers existed in the arrival measurements. The basic process of the KDE method is described as follows. Six sensors are randomly selected from the positioning system with n(n>6) sensors, and the initial AE source coordinates are obtained by solving Equation (5). For a positioning system with *n* sensors, there will be $C_{n}^{6}$ sensor combinations to generate $C_{n}^{6}$ sets of algebraic solutions. After filtering the imaginary solutions, m groups of initial source coordinates can be obtained. Then, the tri-variate kernel density estimator is used to fit these initial solutions. The tri-variate kernel density estimator of the unknown density *f* can be constructed as

(6) $$f\left(\theta \right)=\frac{1}{m}{\left|H\right|}^{\frac{1}{2}}\overset{m}{\underset{i=1}{\sum }}K\left(H^{-\frac{1}{2}}\left(\theta -\theta_{i}\right)\right)$$

where τ is a tri-variate random vector of the density function *f*, where the superscript *T* represents transposition; $\theta_{i}$ is a tri-variate random sample from the density function *f* for i=1,2,⋯,m; $K\left(\cdot \right)$ is the tri-variate kernel function; H is the 3×3 diagonal bandwidth matrix, and its each element is

(7) $$H_{ii}=\sigma_{i}^{2}{\left(\frac{4}{5m}\right)}^{\frac{2}{7}},\;\;i=1,2,3$$

and $H_{ij}=0$, i=j; $\sigma_{i}$ is the scale parameter of the *i*th variable. 

From the above processes, the density function *f* of the AE source coordinate can be determined, and the AE source coordinate corresponding to the extreme value of the density function is deemed as the final solution. The results showed that the minimum and maximum absolute distance errors of the KDE method were 6.16 and 9.92 mm, respectively, while the errors of the method of Dong et al. [20,35] were 12.34 and 71.58 mm. The positioning accuracy of this method was further improved; however, it is still not optimal under the random measurement errors (no outliers). The reason behind this is that the initial localization uses only a minimum number of six sensors, which is mathematically required for pin-point localization without optimization ability; therefore, the initial localization is so sensitive to measurement error that a small error will result in a large initial location deviation. Although the tri-variate kernel density estimator is further used to fit these initial location results, the optimal AE source coordinate in a statistical sense is still difficult to obtain by extracting the information from these poor initial location results. Moreover, the real-time application of this method is rapidly reduced with the increase in the number of sensors  n, because the calculation times $C_{n}^{6}$ of the initial positioning will promptly increase. In order to avoid intensive initial positioning and alleviate the computational burden, this paper comprehensively utilizes all the sensors in a single calculation. Moreover, to better utilize all sensors and yield a more accurate positioning result, the equation residuals of the linear TDOA equations are calculated and then minimized. The detailed derivation of the proposed method is depicted in the following section.

## 3. Theory of the Proposed Method

### 3.1. Transform the Nonlinear Hyperboloid Equations into Linear Ones 

**Figure 1:**
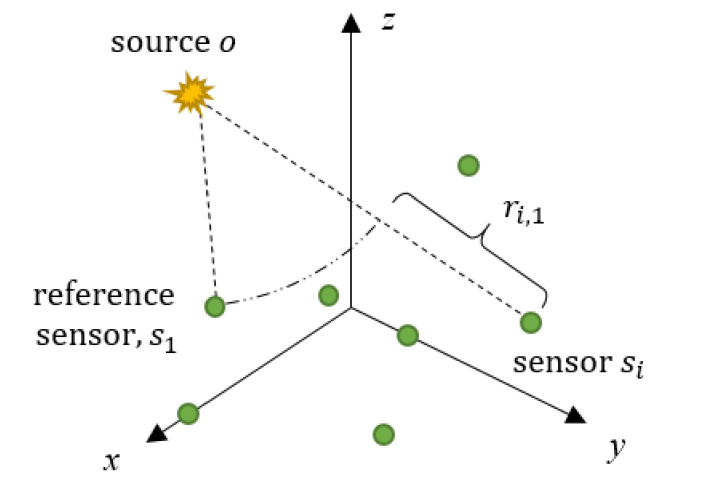
Source and sensors layout in a monitoring system.

Let us reconsider a monitoring system with $n\left(n\geq 6\right)$ sensors. The sensors are numbered as $s_{i}\left(x_{i},\;y_{i},\;z_{i}\right)$, i=1,2,···,n, according to the order in which they receive the AE signals, as shown in Figure 1. The AE source is located at $o\left(x,y,z\right)$. Different from the method based on the arrival measurements described in the previous section, the new method will adopt the TDOA measurements to locate the AE source. To obtain the TDOA measurements, the sensor that first receives the AE signal is determined as reference sensor. Then, all the TDOA measurements are calculated with respect to the reference sensor. In particular, the TDOA measurement between the sensor $s_{i}\left(i=2,3,\cdots ,n\right)$ and the reference sensor $s_{1}$ is expressed as

(8) $$t_{i,1}=t_{i}-t_{1}=\left[\sqrt{{\left(x_{i}-x\right)}^{2}+{\left(y_{i}-y\right)}^{2}+{\left(z_{i}-z\right)}^{2}}-\sqrt{{\left(x_{1}-x\right)}^{2}+{\left(y_{1}-y\right)}^{2}+{\left(z_{1}-z\right)}^{2}}\right]/v\;.$$

The range difference $r_{i,1}$ is the difference between the distances from the source to the sensor $s_{i}\left(i=2,3,\cdots ,n\right)$ and from the source to the reference sensor $s_{1}$, which is in direct proportion to the TDOA $t_{i,1}$ as

(9) $$r_{i,1}=vt_{i,1}=\sqrt{{\left(x_{i}-x\right)}^{2}+{\left(y_{i}-y\right)}^{2}+{\left(z_{i}-z\right)}^{2}}-\sqrt{{\left(x_{1}-x\right)}^{2}+{\left(y_{1}-y\right)}^{2}+{\left(z_{1}-z\right)}^{2}}.$$

**Figure 2:**
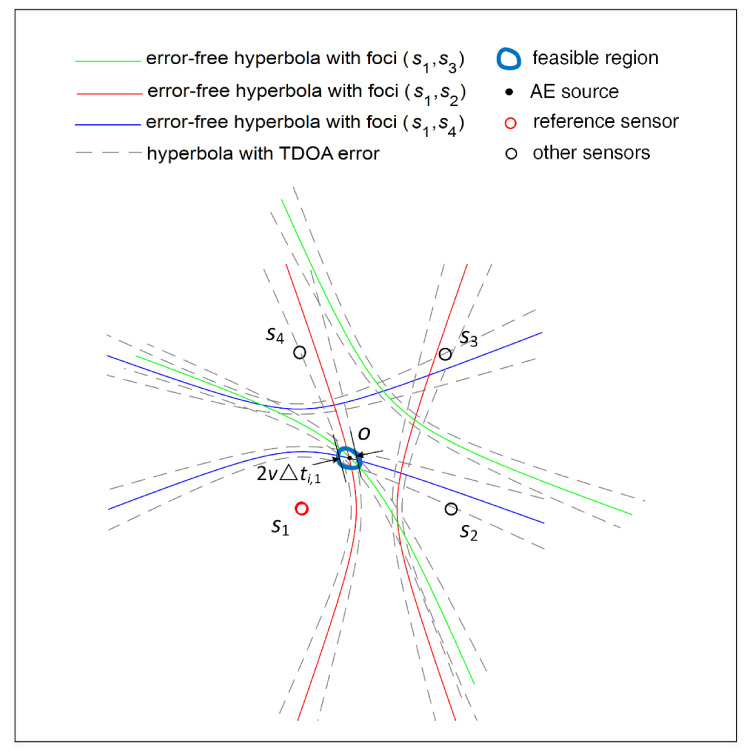
The hyperbolic curves determined by TDOA measurements.

The above equation defines a hyperboloid of two sheets with the foci of $\left(s_{1},\;s_{i}\right)$ and major axis length of $r_{i,1}$. Naturally, for a two-dimensional AE positioning system, Equation (9) will determine a hyperbola with the foci of $\left(s_{1},\;s_{i}\right)$ and major axis length of $r_{i,1}$, as shown in Figure 2. It should be noted that the basic geometric interpretation of the two-dimensional positioning described in Figure 2 are exactly the same as the three-dimensional positioning presented in this paper.

In the absence of TDOA errors, these hyperbolas will intersect at a single point where the AE source is located, as shown in Figure 2. However, the errors are inevitably contained in TDOA measurements in actual scenarios. With the error-containing TDOAs, the highly nonlinear hyperbolas will not exactly intersect at a single point but enclose a feasible region where the AE source may appear. Therefore, we cannot find an exact solution to the position of the AE source, but only a statistical approximate solution. However, due to the high nonlinearity of the hyperbolic equations, it is of great difficulty to find such an approximate solution with the highest probability. An iterative optimization technique has to be used to solve this problem [38,39]. This method starts with an initial guess, and the initial guess is iteratively corrected to find the final AE source coordinate. However, this method faces the difficulties of choosing a close enough initial guess and large iterative calculations. Moreover, its convergence is difficult to guarantee. Therefore, it is necessary to transform the nonlinear hyperboloids in Equation (9) into a set of linear equations. 

To do this, Equation (2) is shifted first and then squared to obtain

(10) $$2x_{i,1}x+2y_{i,1}y+2z_{i,1}z+2t_{i,1}K+t_{i,1}^{2}V-l_{i,1}=0,i=2,\;3,\cdots ,n,$$

where $x_{i,0}=x_{i}-x_{1}$, $y_{i,1}=y_{i}-y_{1}$, $z_{i,1}=z_{i}-z_{1}$, and $K=v\sqrt{{\left(x_{1}-x\right)}^{2}+{\left(y_{1}-y\right)}^{2}+{\left(z_{1}-z\right)}^{2}}$. The intermediate variables K and V make Equation (10) become the linear TDOA equations.

Because the measurements are noisy and the number of sensors is always more than six, Equation (10) is inconsistent. The left and right sides of Equation (10) are not equal, and their deviations are called equation residuals and expressed as

(11) $$\eta_{i}=2x_{i,1}x+2y_{i,1}y+2z_{i,1}z+2t_{i,1}K+t_{i,1}^{2}V-l_{i,1},i=2,\;3,\cdots ,n.$$

### 3.2. Minimize Sum of Squared Residuals with Respect to x, y, and z

Our goal is to find the AE source coordinate that best fits the linear equations, i.e., makes the sum of squared residuals minimal. The sum of the squared equation residuals is expressed as

(12) $$\gamma =\overset{n}{\underset{i=2}{\sum }}\eta_{i}^{2}.$$

To minimize Equation (12), we first take its partial derivatives with respect to the AE source coordinate $\left(x,\;y,\;z\right)$

(13) $$\frac{\partial \gamma }{\partial x}=4\left(a_{1}x+b_{1}y+c_{1}z+d_{1}K+e_{1}V-f_{1}\right),$$

where $a_{1}=\overset{n}{\underset{i=2}{\sum }}x_{i,1}x_{i,1}$, $b_{1}=\overset{n}{\underset{i=2}{\sum }}x_{i,1}y_{i,1}$, $c_{1}=\overset{n}{\underset{i=2}{\sum }}x_{i,1}z_{i,1}$, $d_{1}=\overset{n}{\underset{i=2}{\sum }}x_{i,1}t_{i,1}$, $e_{1}=\frac{1}{2}\overset{n}{\underset{i=2}{\sum }}x_{i,1}t_{i,1}^{2},$ and $f_{1}=\frac{1}{2}\overset{n}{\underset{i=2}{\sum }}x_{i,1}l_{i,1}.$

(14) $$\frac{\partial \gamma }{\partial y}=4\left(a_{2}x+b_{2}y+c_{2}z+d_{2}K+e_{2}V-f_{2}\right),$$

where $a_{2}=\overset{n}{\underset{i=2}{\sum }}y_{i,1}x_{i,1}$, $b_{2}=\overset{n}{\underset{i=2}{\sum }}y_{i,1}y_{i,1}$, $c_{2}=\overset{n}{\underset{i=2}{\sum }}y_{i,1}z_{i,1}$, $d_{2}=\overset{n}{\underset{i=2}{\sum }}y_{i,1}t_{i,1}$, $e_{2}=\frac{1}{2}\overset{n}{\underset{i=2}{\sum }}y_{i,1}t_{i,1}^{2},$ and $f_{2}=\frac{1}{2}\overset{n}{\underset{i=2}{\sum }}y_{i,1}l_{i,1}.$

(15) $$\frac{\partial \gamma }{\partial z}=4\left(a_{3}x+b_{3}y+c_{3}z+d_{3}K+e_{3}V-f_{3}\right),$$

where $a_{3}=\overset{n}{\underset{i=2}{\sum }}z_{i,1}x_{i,1}$, $b_{3}=\overset{n}{\underset{i=2}{\sum }}z_{i,1}y_{i,1}$, $c_{3}=\overset{n}{\underset{i=2}{\sum }}z_{i,1}z_{i,1}$, $d_{3}=\overset{n}{\underset{i=2}{\sum }}z_{i,1}t_{i,1}$, $e_{3}=\frac{1}{2}\overset{n}{\underset{i=2}{\sum }}z_{i,1}t_{i,1}^{2},$ and $f_{3}=\frac{1}{2}\overset{n}{\underset{i=2}{\sum }}z_{i,1}l_{i,1}.$

Then, set these partial derivatives to zeros to obtain the following determined linear equations

(16) $$\left\{\begin{array}{c} a_{1}x+b_{1}y+c_{1}z=-d_{1}K-e_{1}V+f_{1}\\ a_{2}x+b_{2}y+c_{2}z=-d_{2}K-e_{2}V+f_{2}\\ a_{3}x+b_{3}y+c_{3}z=-d_{3}K-e_{3}V+f_{3} \end{array}\right..$$

Finally, the least squares algebraic solution of the AE source coordinate in terms of two intermediates can be calculated as

(17) $$\left\{\begin{array}{c} x=\frac{M_{1}K+N_{1}V+P_{1}}{D}\\ y=\frac{M_{2}K+N_{2}V+P_{2}}{D}\\ z=\frac{M_{3}K+N_{3}V+P_{3}}{D} \end{array}\right.,$$

where D is the determinant with the expression of $D=\left|\begin{array}{ccc} a_{1} & b_{1} & c_{1}\\ a_{2} & b_{2} & c_{2}\\ a_{3} & b_{3} & c_{3} \end{array}\right|$. $M_{j}$, $N_{j}$, and $P_{j}$, for j=1, 2, 3, are also the determinants and like D, but with the *j*th column of D replaced by $\left(-d_{1},\;-d_{2},\;-d_{3}\right)$, $\left(-e_{1},-e_{2},\;-e_{3}\right)$ and $\left(f_{1},\;f_{2},\;f_{3}\right)$, respectively.

However, the AE source coordinate $\left(x,\;y,\;z\right)$ in Equation (17) has not been completely determined, because the two intermediate variables K and V are still unknown and variable. To determine the values of K and V, we substitute Equation (17) into Equation (11) and minimize the sum of squared residuals again, but this time with respect to K and V. In this way, a new system of determined linear equations only about *K* and *V* can be produced, and the solutions of K and V can be easily obtained. After finding the minimizing values of *K* and *V*, they are substituted into Equation (17) again, and the AE source coordinate $\left(x,\;y,\;z\right)$ in Equation (17) automatically becomes the minimizer of the sum of squared residuals [33]. The specific mathematical calculations are shown in the following section. 

### 3.3. Minimize Sum of Squared Residuals with Respect to K and V

Substituting Equation (17) into Equation (11) again, the unknown AE source coordinate (*x*, *y*, *z*) can be eliminated and a new equation residual $\eta_{i}^{\prime }$ with respect to K and V can be obtained as

(18) $$\eta_{i}^{\prime }=p_{i}K+q_{i}V+r_{i},i=2,3,\cdots ,n,$$

where $p_{i}=2\left(\frac{x_{i,1}M_{1}+y_{i,1}M_{2}+z_{i,1}M_{3}}{D}+t_{i,1}\right)$, $q_{i}=2\left(\frac{x_{i,1}N_{1}+y_{i,1}N_{2}+z_{i,1}N_{3}}{D}+\frac{1}{2}t_{i,1}^{2}\right)$, and $r_{i}=2\left(\frac{x_{i,1}P_{1}+y_{i,1}P_{2}+z_{i,1}P_{3}}{D}-\frac{1}{2}l_{i,1}\right)$.

The corresponding sum of squared residuals is given by

(19) $$\gamma^{\prime }=\overset{n}{\underset{i=2}{\sum }}{\left(\eta_{i}^{\prime }\right)}^{2}.$$

Take the partial derivatives of Equation (19) with respect to *K* and *V*, and this yields

(20) $$\frac{\partial \gamma^{\prime }}{\partial K}=2\left(u_{1}K+w_{1}V-k_{1}\right),$$

where $u_{1}=\overset{n}{\underset{i=2}{\sum }}p_{i}p_{i}$, $w_{1}=\overset{n}{\underset{i=2}{\sum }}p_{i}q_{i}$, and $k_{1}=-\overset{n}{\underset{i=2}{\sum }}p_{i,1}r_{i,1}.$

(21) $$\frac{\partial \gamma^{\prime }}{\partial V}=2\left(u_{2}K+w_{2}V-k_{2}\right),$$

where $u_{2}=\overset{n}{\underset{i=2}{\sum }}q_{i}p_{i}$, $w_{2}=\overset{n}{\underset{i=2}{\sum }}q_{i}q_{i}$, and $k_{2}=-\overset{n}{\underset{i=2}{\sum }}q_{i,1}r_{i,1}.$

Set the derivatives to zeros, and the determined linear equations about *K* and *V* will be

(22) $$\left\{\begin{array}{c} u_{1}K+w_{1}V=k_{1}\\ u_{2}K+w_{2}V=k_{2} \end{array}\right.$$

By solving Equation (22), the two intermediate variables *K* and *V* can be solved as

(23) $$\left\{\begin{array}{c} K=\frac{M_{1}^{'}}{D^{\prime }}\\ V=\frac{M_{2}^{'}}{D^{\prime }} \end{array}\right.,$$

where $D^{\prime }$, $M_{1}^{\prime }$, and $M_{2}^{\prime }$ are the determinants given by $D^{\prime }=\left|\begin{array}{cc} u_{1} & w_{1}\\ u_{2} & w_{2} \end{array}\right|$, $M_{1}^{\prime }=\left|\begin{array}{cc} k_{1} & w_{1}\\ k_{2} & w_{2} \end{array}\right|$, and $M_{2}^{\prime }=\left|\begin{array}{cc} u_{1} & k_{1}\\ u_{2} & k_{2} \end{array}\right|$, respectively.

**Figure 3:**
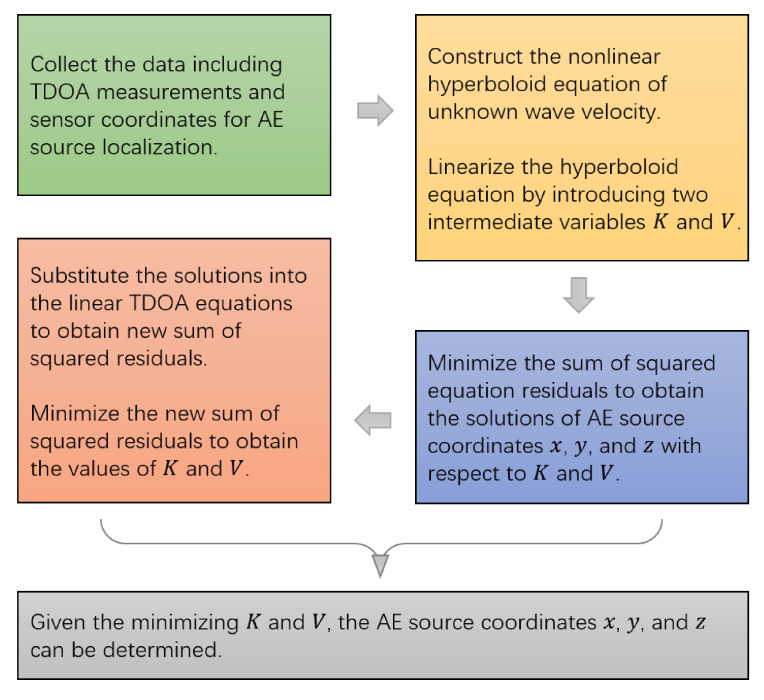
The whole location process of the proposed method.

Substituting the values of *K* and *V* into Equation (17), the final AE source coordinates *x*, *y*, and *z* can be drawn as

(24) $$\left\{\begin{array}{c} x=\frac{M_{1}\left(\frac{M_{1}^{\prime }}{D^{\prime }}\right)+N_{1}\left(\frac{M_{2}^{\prime }}{D^{\prime }}\right)+P_{1}}{D}\\ y=\frac{M_{2}\left(\frac{M_{1}^{\prime }}{D^{\prime }}\right)+N_{2}\left(\frac{M_{2}^{\prime }}{D^{\prime }}\right)+P_{2}}{D}\\ z=\frac{M_{3}\left(\frac{M_{1}^{\prime }}{D^{\prime }}\right)+N_{3}\left(\frac{M_{2}^{\prime }}{D^{\prime }}\right)+P_{3}}{D} \end{array}\right.,$$

which minimizes the sum of squared equation residuals. The whole procedure of the proposed method is shown in Figure 3.

## 4. The Experiment of Pencil-Lead Breaks

**Figure 4:**
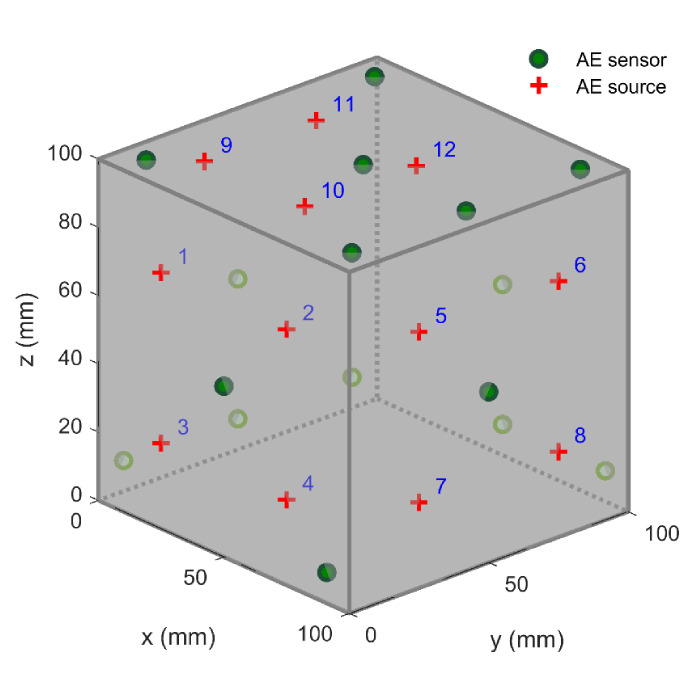
Layout of AE sensors and sources in the experiment of pencil-lead breaks.

**Figure 5:**
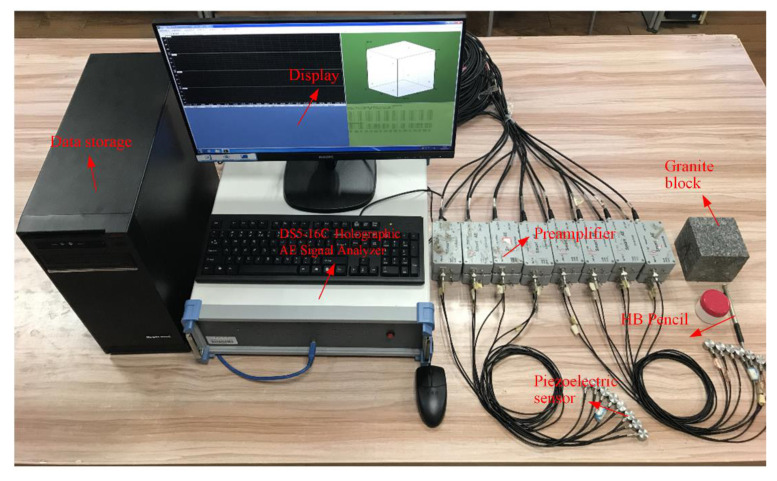
The experimental equipment for collecting the AE signal generated by pencil-lead breaks.

To investigate if the proposed method formulated above can improve the positioning accuracy, the proposed method was applied to the pencil-lead breaks experiment carried out on a 100 × 100 × 100 mm granite block, as shown in Figure 4. The boundaries of cubic granite were relatively flat and smooth without an obvious depression and bulge. Sixteen AE sensors were attached on the surfaces of the cubic granite, and as scattered as possible to guarantee a good geometry array. Moreover, to ensure that the sensor was closely coupled with the granite surface, we smeared an appropriate amount of coupling agent on the sensor and fixed it on the granite surface with adhesive tape. The coordinates of the 16 sensors were (0, 91, 9), (0, 9, 9), (9, 91, 100), (9, 9, 100), (91, 0, 9), (91, 9, 100), (91, 91, 100), (91, 100, 9), (50, 50, 100), (0, 50, 50), (50, 0, 50), (100, 50, 50), (50, 100, 50), (50, 100, 9), (0, 50, 9), and (91, 50, 100), respectively. A broken pencil lead produces the acoustic waves, and the location where the pencil lead breaks is the location of the AE source. The HB pencil lead with a diameter of 0.5 mm was adopted in this experiment and broke at an angle of 30 degrees to the surface of the block. Twelve AE sources were chosen, and their coordinates can be seen in Table 1. The acoustic waves were detected by piezoelectric sensors with the main frequency range from 50 to 400 Hz. The acoustic waves then were amplified by a pre-amplifier with the gain of 40 dB. After that, they were collected by a DS5-16C Holographic AE Signal Analyzer with the sampling frequency of 3 MHz. Finally, the arrival times were picked using the threshold method embedded in the signal analyzer, and the threshold was set to 10 mv, which was slightly higher than the noise. The experimental equipment for collecting the AE signal is shown in Figure 5. After obtaining the arrival times of each sensor, the sensors were numbered as $s_{i}\;\left(i=1,2,\cdots ,16\right)$ according to the ascending order of arrival times, and the sensor that received the signal first was deemed the reference sensor $s_{1}$. Furthermore, the TDOA measurements could be obtained by subtracting the arrival time of the reference sensor $s_{1}$ from the arrival times of other sensors $s_{i}\;\left(i,=2,\;3,\cdots ,16\right)$. In this experiment, no additional errors were added to the TDOA measurements. Therefore, the errors contained in the TDOA measurements were mainly caused by the environmental noise and the arrival picking process.

**Figure 6:**
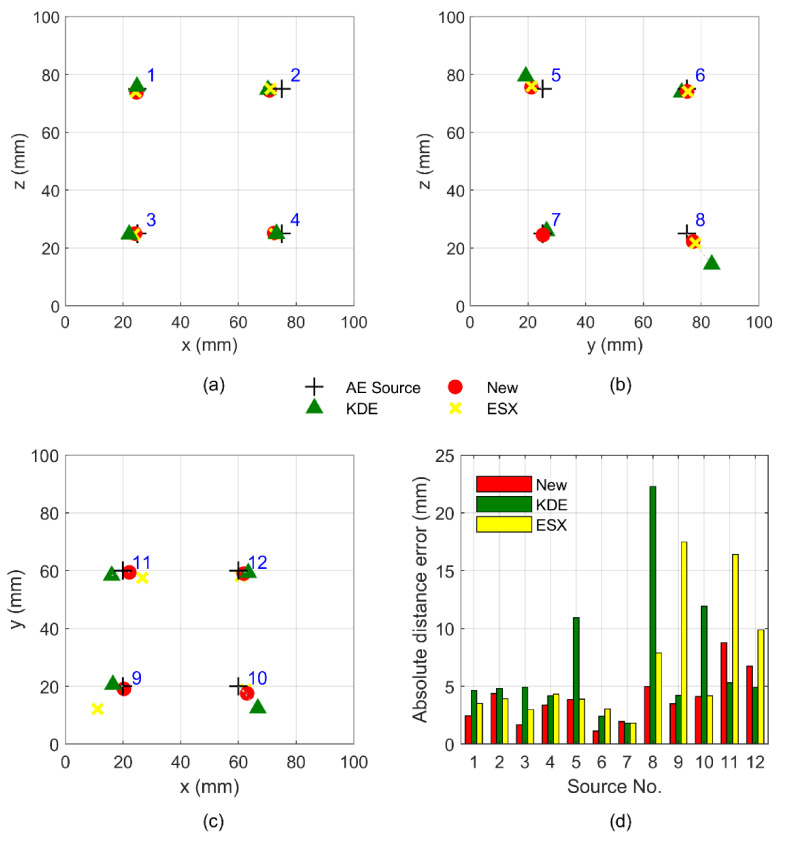
Upper graphs compare the location deviation of new method with that of the extended principle of spherical intersection (ESX) and kernel density estimator (KDE) methods at sources No.1–12; graphs (**a**), (**b**), and (**c**) show the projections of the AE sources on the x–z, y–z, and x–y planes, while graph (**d**) shows the absolute distance errors of the AE sources.

According to the TDOA measurements and sensor coordinates, the locations of 12 AE sources can be efficiently determined by Equation (24). The detailed AE source coordinates solved by the proposed method are listed in Table 1. The best location accuracy of the new method is 1.12 mm, while the best location accuracies of ESX and KDE methods are greater, at 1.80 and 1.82 mm, respectively. At the same time, the maximum absolute distance error of the new method is 8.76 mm, which is far less than the errors of ESX (17.49 mm) and KDE methods (22.27 mm). These location results of the 12 AE sources and their average absolute distance error are illustrated in Figure 6. In this figure, the plus marker “+” denotes the position of the true source, while the circular marker “●”, triangle marker “▲” and cross maker “×” represent the location results of the new method, ESX method, and KDE method, respectively. From Figure 6a–c, we can observe that the circular marker “●” is generally closer to the plus marker “+” than the triangle marker “▲” and cross marker “×”. This means that the location accuracy of the new method is always higher than that of the two traditional methods. Figure 6d quantitatively describes the positioning deviations of the proposed method and compares it with the two traditional methods. We can observe that the absolute distance errors (4.37 and 1.94 mm) of the new method at sources No. 2 and No. 7 are greater than those of the ESX method (3.91 and 1.80 mm). At the same time, the new method also has greater absolute distance errors (1.94, 8.76, and 6.73 mm) at sources No. 7, 11, and 12 than the KDE method (1.82, 5.31, and 4.88 mm), whereas, for most other AE sources, the absolute distance errors of the new method are smaller than those of the ESX and KDE methods. Therefore, the proposed method always has a better positioning performance than the traditional methods.

**Figure 7:**
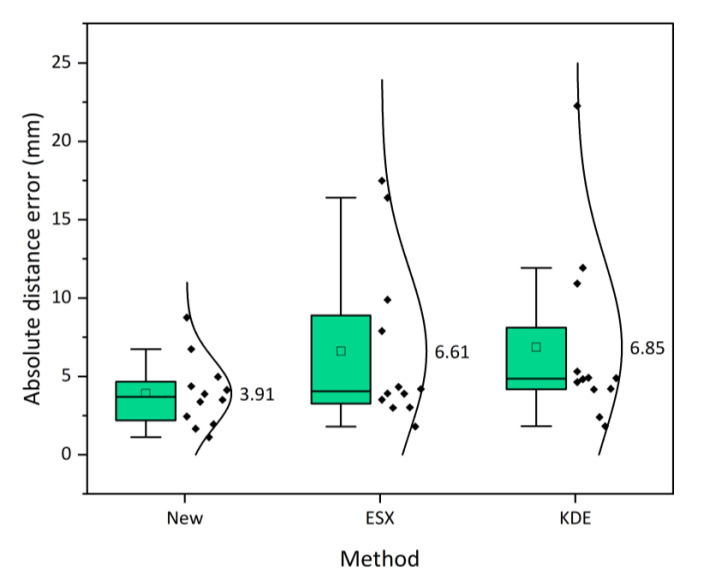
Half-violin diagram for the absolute distance errors of 12 AE sources determined by new, ESX and KDE methods. The values on the right of the diagram denote the corresponding average absolute distance errors.

To further study the distribution condition and probability density of the positioning errors of the 12 AE sources, a half-violin diagram is plotted, as shown in Figure 7. Half of this figure is a dot diagram arranged in intervals of the absolute distance errors, and the other half is a corresponding violin diagram. It can be seen that the error distribution of the new method is more uniform, and the extreme value of the probability density is higher than those of the ESX and KDE methods. Therefore, the proposed method holds a more stable positioning accuracy than the two traditional methods. In addition, the average absolute distance error (3.91 mm) corresponding to the probability density extremum of the new method is less than the ESX method (6.61 mm) and KDE method (6.85 mm). Therefore, compared with the ESX and KDE methods, the positioning accuracy of the proposed method is improved by 40.88% and 43.00%, respectively.

## 5. Simulation Analysis

Due to the existence of environmental noises, the TDOA measurements inevitably contain errors. Therefore, it is necessary to study the positioning accuracy of the proposed method under different TDOA errors. However, the TDOA errors in the pencil-lead breaks experiment in Section 4 are uncontrollable, and quantitative analysis of the influence of the TDOA errors is difficult to carry out. Moreover, the AE sources can only be generated on the surface of the granite block in the pencil-lead breaks experiment; therefore, the positioning performances of the AE sources inside and outside the sensor array are still not clear. Finally, the repeatability of the pencil-lead breaks experiment is poor, and it is not easy to generate a large amount of experimental data in one positioning. For these reasons, three-part simulation tests were carried out to further verify the positioning performance of the proposed method.

**Figure 8:**
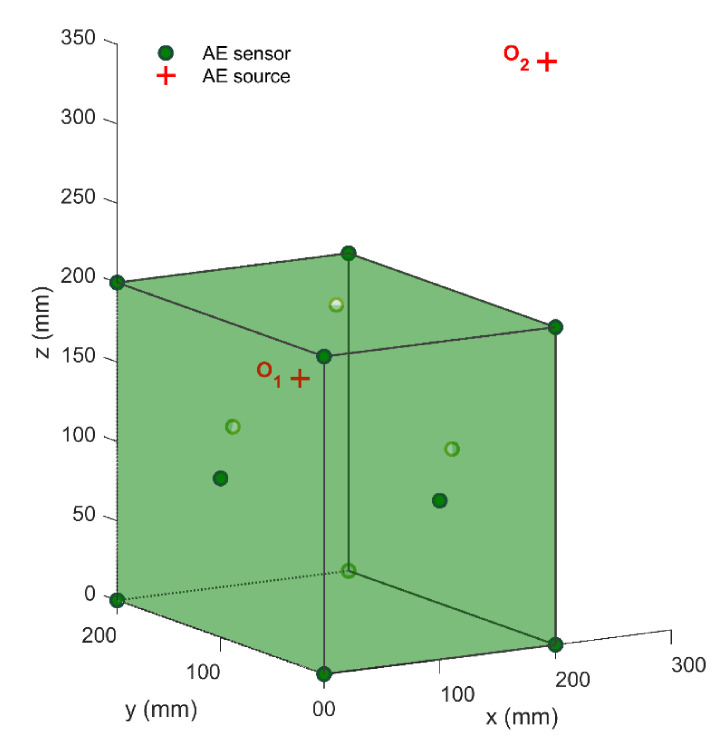
Schematic diagram of virtual monitoring system.

Herein, we established a cubic positioning system with a side length of 200 mm, as shown in Figure 8. Thirteen AE sensors *s_i_* (*i* = 1, 2, ⋯, 13) were located at (0, 0, 0), (200, 0, 0), (200, 200, 0), (0, 200, 0), (0, 0, 200), (200, 0, 200), (200, 200, 200), (0, 200, 200), (100, 0, 100), (200, 100, 100), (100, 200, 100), (0, 100, 100), and (100, 100, 200) (in mm), respectively. In this simulation, the media outside and inside the monitoring system were the same. The true wave velocity of this virtual positioning system was set to 5000 m/s to generate the virtual arrival times. However, the wave velocity was still treated as an unknown in the source positioning process. Then, the virtual TDOA measurements can be formed by subtracting the arrival time of the reference sensor $s_{1}$ from the arrival times of other sensors *s_i_* (*i* = 2, 3, ⋯, 13). Finally, according to the error-containing TDOA measurements and sensor coordinates, the AE source coordinates could be determined efficiently. The positioning results of the proposed method were collected by simulation tests, repeated 100 times and compared with those of ESX, and KDE methods.

**Figure 9:**
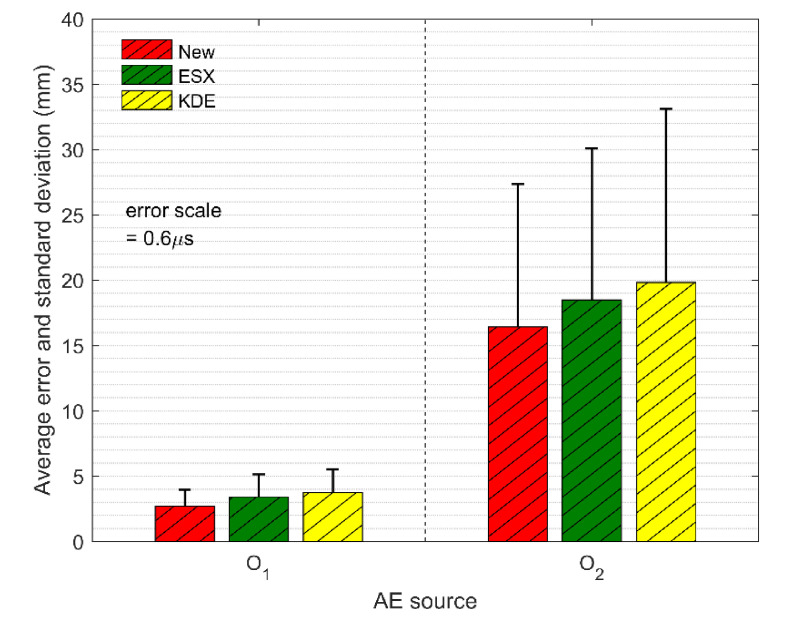
The comparison of the average absolute distance errors and the standard deviations of three methods at TDOA errors of 0.6 μs.

The first part of the simulation test was to verify the location accuracies of the AE sources inside and outside the sensor array. To do this, we set up two virtual AE sources in this simulation test: one was set at $o_{1}$ (200, 100, 200 mm) inside the positioning system, and the other was set at $o_{2}$ (500, 200, 200 mm) outside the positioning system. Moreover, to simulate the influence caused by small environment noise, the random errors with the standard deviation of 0.6 μs were added to the TDOA measurements. The comparisons of location errors in the AE sources inside and outside the sensor array are shown in Figure 9. It can be seen that the average absolute distance errors of all three methods at the source $o_{1}$ are less than 4 mm, while the average absolute distance errors at the source $o_{2}$ are more than 15 mm. Besides, the standard deviations of the location results at the AE source $o_{1}$ are also significantly lower than those at the AE source $o_{2}$. Therefore, the location accuracy and the stability of the AE source inside the sensor array are both higher than those outside the sensor array. The reason behind this is that the non-uniform distribution of the hyperbolic field for the AE source position leads to the non-uniform amplification effect of the sensor array on the TDOA errors. Concretely speaking, because of the existence of TDOA errors, the calculated AE source falsely falls on the adjacent hyperbola. The distance between two hyperbolas is the positioning error. The hyperbolic density outside the sensor array is far lower than that inside the sensor array, which means that the two adjacent hyperbolas outside the sensor array have a longer distance. Therefore, the positioning error of the AE source outside the sensor array is, in general, greater than that inside the sensor array [40]. In this paper, it is suggested that the sensor layout should surround the monitored area as much as possible.

**Figure 10:**
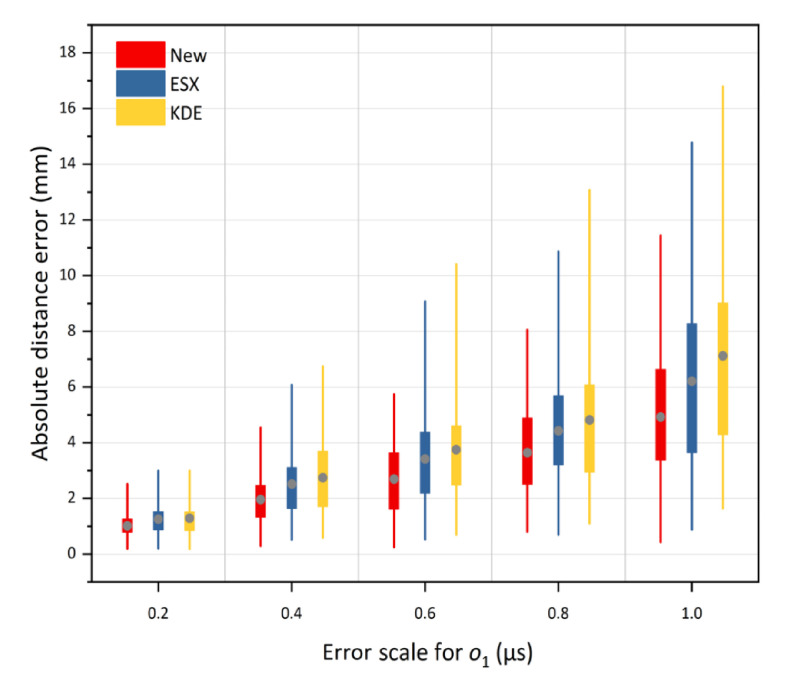
The average absolute distance errors of the new method under TDOA errors of 0.2 μs to 1.0 μs at inside source $o_{1}$, versus KDE and ESX methods.

**Figure 11:**
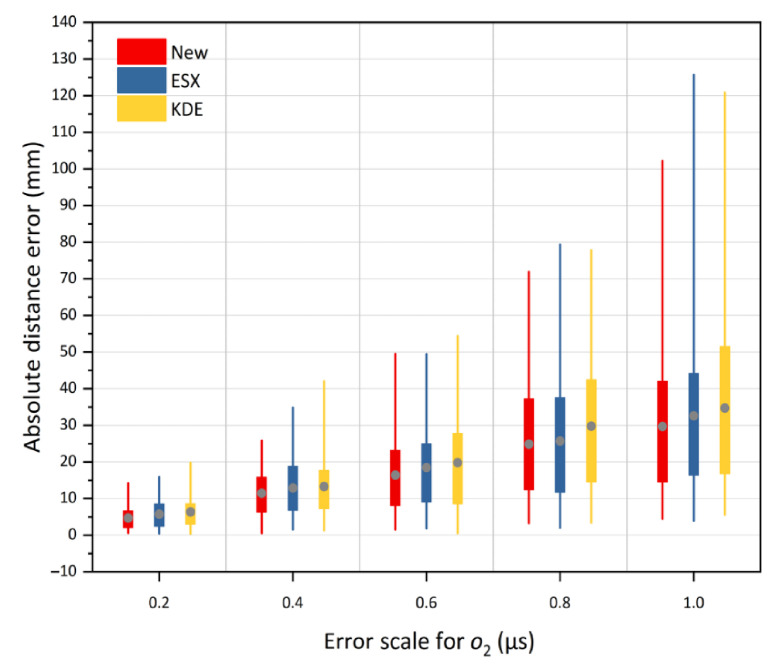
The average absolute distance errors of the new method under TDOA errors of 0.2 μs to 1.0 μs at outside source $o_{2}$, versus KDE and ESX methods.

The second part of the simulation test was to verify the location accuracy of the proposed method under different TDOA errors. The random errors with the standard deviations of 0.2, 0.4, 0.6, 0.8, and 1.0 μs were added to the TDOA measurements. The simulation results at source $o_{1}$ and $o_{2}$ are shown in Figure 10 and Figure 11, respectively. For each box in the two Figures, the position of the grey solid circle denotes the average absolute distance error, and the box extends vertically between the lower quartile and upper quartile of 100 absolute distance errors. The change tendencies of the location results shown in Figure 10 and Figure 11 are similar. We can observe that with an increase in the error scale, the positions of the solid circles for all the three methods continue to rise, which indicates the increase in the location errors of all three methods. In addition, the lengths of the box and the extension line for the three methods are enlarged with the ascending error scale, indicating a decrease in the location stability. However, under any given error scales, both the positioning accuracy and stability of the proposed method are higher than those of the traditional methods. Therefore, the proposed method always has a better tolerance of the TDOA error. This is because this method has more reasonable optimization criteria, which can make full use of all the TDOA measurements to obtain the statistically optimal solution in least squares significance.

**Figure 12:**
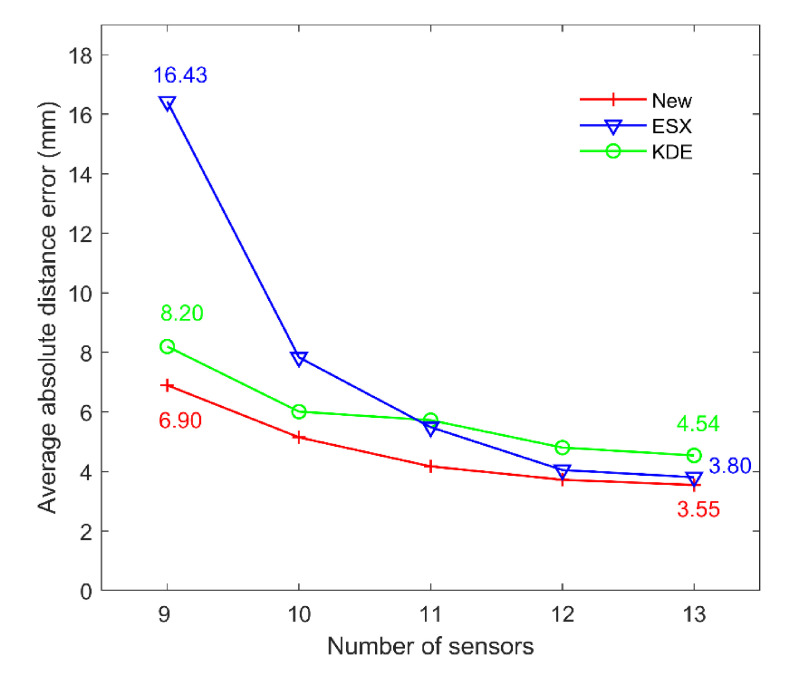
Comparisons of the average absolute errors of three methods under different numbers of sensors.

The third part of the simulation test was to study the influence of the number of sensors on the location accuracy. A total of 9 to 13 sensors were selected from the monitoring system to estimate the AE source coordinates. The error scale was set to 0.6 μs to simulate the influence caused by the environment noise. Moreover, in order to better verify the performance of the proposed method, 100 virtual AE sources inside the sensor array were randomly generated to perform the localization. The average absolute distance errors solved by three methods under different number of sensors are compared in Figure 12. We can observe that different numbers of sensors have different effects on different positioning methods. However, the new method can always maintain the best positioning performance under different numbers of sensors, compared to the traditional methods. Moreover, with the increase in the number of sensors, the positioning error of the proposed method decreases from 6.90 to 3.55 mm, the positioning error of ESX method decreases from 16.43 to 3.80 mm, and the positioning error of KDE method decreases from 8.20 to 4.54 mm. Therefore, we can find that only four sensors are added to the positioning system, and the positioning accuracy of the new method, ESX method and KDE method is improved by 46.0%, 75.4% and 41.1%, respectively. However, it should be noted that when the number of sensors is above 12, the positioning accuracy gradually tends to become stable. Therefore, in the actual positioning system, we suggest that at least 12 sensors are used to ensure the positioning accuracy, and these 12 sensors should be scattered as far around the monitoring area as possible.

## 6. Conclusions

In this paper, an algebraic solution of AE source localization without premeasuring the wave velocity is proposed. This method introduces two intermediate variables to construct the linear TDOA equations of unknown wave velocity, and then obtains the algebraic solution of the AE source by minimizing the residual sum of the linear TDOA equations. The proposed method highlights the following advantages: (1) the average wave velocity can be inversed in real-time, avoiding the influence of measurement error in wave velocity on the localization accuracy; (2) only small determinants are calculated for the final solution, which avoids the difficulty of solving the pseudo-inverse of singular matrix; (3) a more accurate algebraic solution is obtained by efficiently fusing all the TDOA measurements by a single calculation; (4) this method gives a unique algebraic solution, and there is no case of no solution and multiple solutions; (5) the real-time application is favorable, because it avoids the initial guess and the convergence difficulties. The experiment of pencil-lead breaks verifies that the best location accuracy of the proposed method reaches 1.12 mm, which is higher than those of the ESX method (1.80 mm) and KDE method (1.82 mm). Moreover, compared with the ESX and KDE methods, the average positioning errors of the proposed method are reduced by 40.88% and 43.00% respectively. The simulation tests under different TDOA errors indicate that with the increase of the TDOA errors, the positioning errors of both the inside and outside AE sources increase, but the positioning accuracy of the inside AE source is generally higher than that of the outside AE source; furthermore, no matter whether the AE source is inside or outside the sensor array, the proposed method always has a better location performance under any given TDOA errors. The simulation tests under different numbers of sensors further prove that with the increase in the number of sensors, the positioning accuracy of the proposed method will be further improved by 46%; meanwhile, the proposed method always has the highest positioning accuracy compared with the traditional methods under any given number of sensors.

Actually, the proposed method still has the following limitations: (1) there will be a large error in locating the AE source in the medium with strong anisotropy, because the differences among the wave velocities of different directions are disregarded in this method; (2) the proposed method assumes that the wave velocity travels along a straight line, which is not suitable for the scenario where the AE signal travels along the refraction path or other more complicated paths; (3) when outliers exist in the TDOA measurements, the proposed method will still have a large deviation in the location result. Therefore, it is necessary to conduct further research on these limitations.

## Figures and Tables

**Table 1:** The location results of 12 AE sources determined by different methods ^1^.

Source No.	Method	Coordinates			Error	Source No.	Method	Coordinates			Error
		X	Y	Z				X	Y	Z	
1	true	25.00	0.00	75.00	-	7	true	100.00	25.00	25.00	-
	New	24.71	−2.09	73.77	2.44		New	101.87	25.21	24.52	1.94
	ESX	24.46	−3.33	74.02	3.51		ESX	101.73	25.23	24.55	1.80
	KDE	24.84	−4.56	75.80	4.63		KDE	99.40	26.46	25.90	1.82
2	true	75.00	0.00	75.00	-	8	true	100.00	75.00	25.00	-
	New	70.76	0.89	74.46	4.37		New	103.49	77.22	22.24	4.97
	ESX	71.09	0.14	74.96	3.91		ESX	106.54	78.00	21.75	7.89
	KDE	70.20	0.32	74.72	4.82		KDE	117.52	83.66	14.32	22.27
3	true	25.00	0.00	25.00	-	9	true	20.00	20.00	100.00	-
	New	24.18	−1.44	24.96	1.66		New	20.31	19.10	96.63	3.50
	ESX	23.83	−2.74	24.76	2.99		ESX	11.15	12.16	112.88	17.49
	KDE	22.15	−3.99	24.70	4.91		KDE	16.49	20.56	102.23	4.20
4	true	75.00	0.00	25.00	-	10	true	60.00	20.00	100.00	-
	New	72.35	−2.09	25.19	3.38		New	63.02	17.52	98.66	4.13
	ESX	72.61	−3.60	24.92	4.33		ESX	62.52	18.82	96.87	4.19
	KDE	73.18	−3.75	24.93	4.17		KDE	66.74	12.38	106.22	11.93
5	true	100.00	25.00	75.00	-	11	true	20.00	60.00	100.00	-
	New	99.54	21.20	75.57	3.87		New	22.18	59.41	91.54	8.76
	ESX	101.02	21.35	75.90	3.89		ESX	26.64	57.57	85.21	16.39
	KDE	108.20	19.25	79.35	10.92		KDE	16.05	58.31	96.88	5.31
6	true	100.00	75.00	75.00	-	12	true	60.00	60.00	100.00	-
	New	100.68	75.1	74.12	1.12		New	61.86	58.99	93.61	6.73
	ESX	102.84	75.42	74.07	3.02		ESX	60.82	58.13	90.33	9.88
	KDE	98.92	73.24	73.79	2.40		KDE	63.42	59.25	96.60	4.88

^1^ All data in this table are in mm, and the Error refers to the absolute distance error.

## Data Availability

Not applicable.
